# Supplementary material for: Association between normal weight obesity and comorbidities and events of cardiovascular diseases among adults in South China
Source: PLoS One. 2025 Jan 8;20(1):e0316346. doi: 10.1371/journal.pone.0316346 (PMC11709311; doi:10.1371/journal.pone.0316346)
Supplement: S1 File — (DOCX) [file pone.0316346.s001.docx]

6,307 individual participants completed the survey

Participants excluded:

Data on BMI and BF% data were missing (*N* = 63)

BF% below 5% (*N* = 4)

BMI over 24 kg/m^2^ (*N* = 2,796)

BMI below 18.5 kg/m^2^ (*N* = 358)

3,086 normal BMI participants were included for analysis：

Male (*N* = 1,218); Female (*N* = 1,868)

**Supplementary Figure 1. Flow chart of the subject selection.** BF%, body fat percentage; BMI, body mass index; *N*, number of subjects.

**Supplementary Table 1.** **Prevalence (%) of** **abdominal** **obesity, diabetes, hypertension, dyslipidemia, metabolic syndrome, chronic kidney diseases, and cardiovascular diseases in the total, male, and female populations among different** **tertile BF% groups.**

|  | Low | Medium | High | Total | *P* |
| --- | --- | --- | --- | --- | --- |
| Total |  |  |  |  |  |
| AO | 14 (1.36) | 51 (4.91) ^a^ | 100 (9.79) ^a, b^ | 165 (5.35) | < 0.001 |
| Diabetes | 28 (2.74) | 51 (4.93) ^a^ | 58 (5.68) ^a^ | 137 (4.45) | 0.004 |
| E-HTN | 99 (9.68) | 137 (13.21) ^a^ | 194 (19.04) ^a, b^ | 430 (13.97) | < 0.001 |
| H-HTN | 85 (8.34) | 118 (11.41) | 169 (16.58) ^a, b^ | 372 (12.11) | < 0.001 |
| Dyslipidemia | 188 (18.31) | 266 (25.63) ^a^ | 324 (31.73) ^a, b^ | 778 (25.21) | < 0.001 |
| MetS | 11 (1.08) | 40 (3.86) ^a^ | 69 (6.76) ^a, b^ | 120 (3.90) | < 0.001 |
| CKD | 6 (0.59) | 10 (0.97) | 21 (2.06) ^a^ | 37 (1.20) | 0.007 |
| CVD | 18 (1.75) | 36 (3.47) ^a^ | 50 (4.90) ^a^ | 104 (3.37) | < 0.001 |
| Male |  |  |  |  |  |
| AO | 4 (0.99) | 14 (3.44) | 43 (10.54) ^a, b^ | 61 (5.01) | < 0.001 |
| Diabetes | 12 (2.99) | 29 (7.14) ^a^ | 31 (7.60) ^a^ | 72 (5.92) | 0.009 |
| E-HTN | 47 (11.66) | 74 (18.18) ^a^ | 104 (25.49) ^a, b^ | 225 (18.47) | < 0.001 |
| H-HTN | 43 (10.70) | 70 (17.24) ^a^ | 101 (24.75) ^a, b^ | 214 (17.60) | < 0.001 |
| Dyslipidemia | 85 (21.09) | 141 (34.64) ^a^ | 173 (42.40) ^a^ | 399 (32.76) | < 0.001 |
| MetS | 3 (0.75) | 22 (5.41) ^a^ | 43 (10.54) ^a, b^ | 68 (5.59) | < 0.001 |
| CKD | 3 (0.75) | 4 (0.99) | 13 (3.19) ^a^ | 20 (1.64) | 0.011 |
| CVD | 10 (2.48) | 17 (4.18) | 26 (6.37) ^a^ | 53 (4.35) | 0.024 |
| Female |  |  |  |  |  |
| AO | 10 (1.60) | 37 (5.86) ^a^ | 57 (9.30) ^a^ | 104 (5.57) | < 0.001 |
| Diabetes | 16 (2.58) | 22 (3.50) | 27 (4.40) | 65 (3.49) | 0.216 |
| E-HTN | 52 (8.39) | 63 (10.00) | 90 (14.73) ^a, b^ | 205 (11.02) | 0.001 |
| H-HTN | 42 (6.81) | 48 (7.64) | 68 (11.13) ^a^ | 158 (8.51) | 0.016 |
| Dyslipidemia | 103 (16.51) | 125 (19.81) | 151 (24.63) ^a^ | 379 (20.29) | 0.002 |
| MetS | 8 (1.29) | 18 (2.86) | 26 (4.24) ^a^ | 52 (2.79) | 0.007 |
| CKD | 3 (0.48) | 6 (0.95) | 8 (1.31) | 17 (0.91) | 0.313 |
| CVD | 8 (1.28) | 19 (3.01) | 24 (3.92) ^a^ | 51 (2.73) | 0.015 |

AO, abdominal obesity; CKD, chronic kidney disease; CVD, cardiovascular diseases; E-HTN, essential hypertension; H-HTN, H-type hypertension; MetS, metabolic syndrome.

All subjects represented have a BMI of 18.5 to 24.0 kg/m^2^ and are stratified according to tertile [male: tertile boundaries were as low (BF% < 20.5%), medium (20.5% ≤ BF% < 24.6%) and high (BF% ≥ 24.6%); female: low (BF% < 29.9%), medium (29.9% ≤ BF% < 33.6%) and high (BF% $\geq$ 33.6%)].

^a^ *P* < 0.05 versus low.

^b^ *P* < 0.05 versus medium.

**Supplementary Table 2.** **Crude and** **adjusted association of normal weight obesity among all subjects with comorbidities and events of CVD.**

|  | Low | Medium | High |
| --- | --- | --- | --- |
| AO |  |  |  |
| Crude OR (95% CI) | Ref 1.00 | 3.74 (2.06 - 6.80) ^***^ | 7.86 (4.46 - 13.84) ^***^ |
| Model 1 (95% CI) | Ref 1.00 | 3.37 (1.85 - 6.15) ^***^ | 6.02 (3.39 - 10.69) ^***^ |
| Model 2 (95% CI) | Ref 1.00 | 3.36 (1.84 - 6.14) ^***^ | 6.05 (3.40 - 10.75) ^***^ |
| Diabetes |  |  |  |
| Crude OR (95% CI) | Ref 1.00 | 1.84 (1.15 - 2.95) ^*^ | 2.14 (1.35 - 3.39) ^**^ |
| Model 1 (95% CI) | Ref 1.00 | 1.45 (0.88 - 2.38) | 1.20 (0.74 - 1.97) |
| Model 2 (95% CI) | Ref 1.00 | 1.32 (0.54 - 3.25) | 2.11 (0.93 - 4.78) |
| E-HTN |  |  |  |
| Crude OR (95% CI) | Ref 1.00 | 1.42 (1.08 - 1.87) ^*^ | 2.20 (1.69 - 2.85) ^***^ |
| Model 1 (95% CI) | Ref 1.00 | 1.15 (0.85 - 1.56) | 1.36 (1.01 - 1.84) ^*^ |
| Model 2 (95% CI) | Ref 1.00 | 1.10 (0.75 - 1.61) | 1.56 (1.09 - 2.22) ^*^ |
| H-HTN |  |  |  |
| Crude OR (95% CI) | Ref 1.00 | 1.42 (1.06 - 1.90) ^*^ | 2.18 (1.66 - 2.88) ^***^ |
| Model 1 (95% CI) | Ref 1.00 | 1.13 (0.82 - 1.57) | 1.30 (0.95 - 1.79) |
| Model 2 (95% CI) | Ref 1.00 | 1.07 (0.73 - 1.58) | 1.43 (0.99 - 2.07) |
| Dyslipidemia |  |  |  |
| Crude OR (95% CI) | Ref 1.00 | 1.54 (1.25 - 1.90) ^***^ | 2.08 (1.69 - 2.55) ^***^ |
| Model 1 (95% CI) | Ref 1.00 | 1.44 (1.16 - 1.79) ^**^ | 1.81 (1.46 - 2.25) ^***^ |
| Model 2 (95% CI) | Ref 1.00 | 1.45 (1.16 - 1.80) ^**^ | 1.85 (1.49 - 2.29) ^***^ |
| MetS |  |  |  |
| Crude OR (95% CI) | Ref 1.00 | 3.69 (1.88 - 7.24) ^***^ | 6.66 (3.50 - 12.66) ^***^ |
| Model 1 (95% CI) | Ref 1.00 | 3.03 (1.52 - 6.01) ^**^ | 4.06 (2.09 - 7.86) ^***^ |
| Model 2 (95% CI) ^a^ | Ref 1.00 | 2.79 (1.37 - 5.70) ^**^ | 4.61 (2.32 - 9.18) ^***^ |
| CKD |  |  |  |
| Crude OR (95% CI) | Ref 1.00 | 1.65 (0.60 - 4.57) | 3.56 (1.43 - 8.86) ^*^ |
| Model 1 (95% CI) | Ref 1.00 | 1.13 (0.40 - 3.22) | 1.42 (0.55 - 3.69) |
| Model 2 (95% CI) | Ref 1.00 | 1.11 (0.38 - 3.22) | 1.47 (0.56 - 3.89) |
| CVD |  |  |  |
| Crude OR (95% CI) | Ref 1.00 | 2.08 (1.11 - 3.87) ^*^ | 3.18 (1.77 - 5.74) ^***^ |
| Model 1 (95% CI) | Ref 1.00 | 1.57 (0.82 - 3.00) | 1.58 (0.85 - 2.95) |
| Model 2 (95% CI) ^b^ | Ref 1.00 | 1.54 (0.81 - 2.96) | 1.56 (0.83 - 2.93) |

AO, abdominal obesity; BF%, body fat percentage; CI, confidence interval; CKD, chronic kidney disease; CVD, cardiovascular disease; E-HTN, essential hypertension; H-HTN, H-type hypertension; MetS, metabolic syndrome; OR, odds ratio; Ref, reference.

All subjects represented had a BMI of 18.5 to 24.0 kg/m^2^ and were stratified according to tertile [male: tertile boundaries were as low (BF% < 20.5%), medium (20.5% ≤ BF% < 24.6%) and high (BF% ≥ 24.6%); female: low (BF% < 29.9%), medium (29.9% ≤ BF% < 33.6%) and high (BF% $\geq$ 33.6%)].

Crude, without adjustment for other risk factors.

Model 1, adjusted for age and gender.

Model 2, adjusted for age, gender, educational level, ethnic group, and statuses of marriage, smoking, and drinking. In addition, the histories of using antidiabetic, antihypertensive, and lipid-regulating drugs were respectively adjusted for diabetes, E-HTN and H-HTN, and dyslipidemia, and the history of using more than three drugs was adjusted for MetS.

^*^ *P* < 0.05, ^**^ *P* < 0.01, and ^***^ *P* < 0.001.

**Supplementary Table 3. Crude and adjusted association of normal weight obesity among male and female subjects with comorbidities and events of CVD.**

| Variables | Male | | |  | Female | | |
| --- | --- | --- | --- | --- | --- | --- | --- |
|  | Low | Medium | High |  | Low | Medium | High |
| AO |  |  |  |  |  |  |  |
| Crude OR (95% CI) | Ref 1.00 | 3.55 (1.16 - 10.89) ^*^ | 11.75 (4.18 - 33.06) ^***^ |  | Ref 1.00 | 3.83 (1.89 - 7.76) ^***^ | 6.30 (3.18 - 12.45) ^***^ |
| Model 1 (95% CI) | Ref 1.00 | 3.22 (1.05 - 9.93) ^*^ | 9.88 (3.47 - 28.11) ^***^ |  | Ref 1.00 | 3.55 (1.73 - 7.27) ^**^ | 4.70 (2.35 - 9.43) ^***^ |
| Model 2 (95% CI) | Ref 1.00 | 2.90 (0.94 - 8.99) | 8.98 (3.14 - 25.70) ^***^ |  | Ref 1.00 | 3.67 (1.78 - 7.55) ^***^ | 4.86 (2.42 - 9.76) ^***^ |
| Diabetes |  |  |  |  |  |  |  |
| Crude OR (95% CI) | Ref 1.00 | 2.50 (1.26 - 4.97) ^**^ | 2.67 (1.35 - 5.28) ^**^ |  | Ref 1.00 | 1.37 (0.71 - 2.64) | 1.74 (0.93 - 3.27) |
| Model 1 (95% CI) | Ref 1.00 | 1.90 (0.92 - 3.91) | 1.48 (0.72 - 3.07) |  | Ref 1.00 | 1.13 (0.57 - 2.24) | 1.01 (0.52 - 1.97) |
| Model 2 (95% CI) | Ref 1.00 | 3.06 (0.81 - 11.63) | 2.45 (0.63 - 9.50) |  | Ref 1.00 | 0.35 (0.07 - 1.81) | 2.21 (0.77 - 6.33) |
| E-HTN |  |  |  |  |  |  |  |
| Crude OR (95% CI) | Ref 1.00 | 1.68 (1.13 - 2.50) ^*^ | 2.59 (1.78 - 3.78) ^***^ |  | Ref 1.00 | 1.21 (0.83 - 1.78) | 1.89 (1.31 - 2.71) ^**^ |
| Model 1 (95% CI) | Ref 1.00 | 1.34 (0.88 - 2.05) | 1.66 (1.10 - 2.52) ^*^ |  | Ref 1.00 | 0.97 (0.62 - 1.52) | 1.11 (0.72 - 1.71) |
| Model 2 (95% CI) | Ref 1.00 | 1.46 (0.89 - 2.39) | 1.66 (1.02 - 2.70) ^*^ |  | Ref 1.00 | 0.81 (0.44 - 1.51) | 1.51 (0.88 - 2.58) |
| H-HTN |  |  |  |  |  |  |  |
| Crude OR (95% CI) | Ref 1.00 | 1.74 (1.16 - 2.62) ^**^ | 2.75 (1.86 - 4.05) ^***^ |  | Ref 1.00 | 1.13 (0.74 - 1.74) | 1.71 (1.15 - 2.56) ** |
| Model 1 (95% CI) | Ref 1.00 | 1.39 (0.90 - 2.16) | 1.77 (1.16 - 2.71) ^**^ |  | Ref 1.00 | 0.88 (0.53 - 1.46) | 0.89 (0.55 - 1.44) |
| Model 2 (95% CI) | Ref 1.00 | 1.54 (0.93 - 2.56) | 1.83 (1.11 - 3.01) ^*^ |  | Ref 1.00 | 0.70 (0.38 - 1.31) | 1.09 (0.62 - 1.92) |
| Dyslipidemia |  |  |  |  |  |  |  |
| Crude OR (95% CI) | Ref 1.00 | 1.98 (1.45 - 2.72) ^***^ | 2.75 (2.02 - 3.75) ^***^ |  | Ref 1.00 | 1.25 (0.94 - 1.67) | 1.65 (1.25 - 2.19) ^***^ |
| Model 1 (95% CI) | Ref 1.00 | 1.83 (1.33 - 2.53) ^***^ | 2.56 (1.86 - 3.53) ^***^ |  | Ref 1.00 | 1.17 (0.87 - 1.57) | 1.37 (1.02 - 1.84) ^*^ |
| Model 2 (95% CI) | Ref 1.00 | 1.85 (1.34 - 2.57) ^**^ | 2.73 (1.97 - 3.79) ^***^ |  | Ref 1.00 | 1.19 (0.88 - 1.61) | 1.38 (1.03 - 1.85) ^*^ |
| MetS |  |  |  |  |  |  |  |
| Crude OR (95% CI) | Ref 1.00 | 7.60 (2.26 - 25.60) ^**^ | 15.67 (4.82 - 50.94) ^***^ |  | Ref 1.00 | 2.25 (0.97 - 5.22) | 3.39 (1.52 - 7.55) ^**^ |
| Model 1 (95% CI) | Ref 1.00 | 6.58 (1.93 - 22.43) ^**^ | 12.35 (3.74 - 40.83) ^***^ |  | Ref 1.00 | 1.86 (0.76 - 4.57) | 1.66 (0.71 - 3.92) |
| Model 2 (95% CI) ^a^ | Ref 1.00 | 7.39 (2.06 - 26.57) ^**^ | 16.36 (4.62 - 57.91) ^***^ |  | Ref 1.00 | 1.61 (0.62 - 4.20) | 2.06 (0.83 - 5.10) |
| CKD |  |  |  |  |  |  |  |
| Crude OR (95% CI) | Ref 1.00 | 1.32 (0.29 - 5.95) | 4.38 (1.24 - 15.48) ^*^ |  | Ref 1.00 | 1.98 (0.49 - 7.97) | 2.72 (0.72 - 10.32) |
| Model 1 (95% CI) | Ref 1.00 | 0.83 (0.18 - 3.87) | 1.78 (0.47 - 6.69) |  | Ref 1.00 | 1.51 (0.36 - 6.37) | 1.18 (0.29 - 4.71) |
| Model 2 (95% CI) | Ref 1.00 | 0.77 (0.16 - 3.70) | 1.65 (0.42 - 6.47) |  | Ref 1.00 | 1.53 (0.35 - 6.69) | 1.27 (0.31 - 5.24) |
| CVD |  |  |  |  |  |  |  |
| Crude OR (95% CI) | Ref 1.00 | 1.50 (0.67 - 3.39) | 2.35 (1.10 - 5.00) ^*^ |  | Ref 1.00 | 3.22 (1.17 - 8.85) ^*^ | 4.83 (1.82 - 12.78) ^*^ |
| Model 1 (95% CI) | Ref 1.00 | 1.04 (0.44 - 2.43) | 1.17 (0.52 - 2.63) |  | Ref 1.00 | 2.74 (0.96 - 7.80) | 2.56 (0.93 - 7.05) |
| Model 2 (95% CI) ^b^ | Ref 1.00 | 0.91 (0.38 - 2.17) | 1.00 (0.44 - 2.30) |  | Ref 1.00 | 2.73 (0.95 - 7.84) | 2.53 (0.91 - 7.06) |

AO, abdominal obesity; BF%, body fat percentage; CI, confidence interval; CKD, chronic kidney disease; CVD, cardiovascular disease; E-HTN, essential hypertension; H-HTN, H-type hypertension; MetS, metabolic syndrome; OR, odds ratio; Ref, reference.

Crude, without adjustment for other risk factors.

Model 1, adjusted for age and gender.

Model 2, adjusted for age, gender, educational level, ethnic group, and statuses of marriage, smoking, and drinking. In addition, the histories of using antidiabetic, antihypertensive, and lipid-regulating drugs were respectively adjusted for diabetes, E-HTN and H-HTN, and dyslipidemia, and the history of using more than three drugs was adjusted for MetS.

^*^ *P* < 0.05, ^**^ *P* < 0.01, and ^***^ *P* < 0.001.
