# Supplementary material for: Association between normal weight obesity and comorbidities and events of cardiovascular diseases among adults in South China
Source: PLoS One. 2025 Jan 8;20(1):e0316346. doi: 10.1371/journal.pone.0316346 (PMC11709311; doi:10.1371/journal.pone.0316346)
Supplement: Attachment — Submitted filename: Response to Reviewers 20240831.docx [file pone.0316346.s003.docx]

Responses are in blue fonts.

Requirements:

Please review your reference list to ensure that it is complete and correct. If you have cited papers that have been retracted, please include the rationale for doing so in the manuscript text, or remove these references and replace them with relevant current references. Any changes to the reference list should be mentioned in the rebuttal letter that accompanies your revised manuscript. If you need to cite a retracted article, indicate the article’s retracted status in the References list and also include a citation and full reference for the retraction notice.

Answer: We have reviewed our reference list, and ensured that it was complete and correct.

Response to Reviewers:

Reviewer #2: I appreciate the authors' responsiveness to my comments; the manuscript has improved. However, there are still several grammatical and structural inconsistencies. To enhance clarity and coherence, I recommend using proof-reading tools to identify and rectify these issues, ensuring the manuscript attains a more polished and professional appearance. Here are a few examples that need revision for better clarity and coherence of the text:

1.Lines 84-88 could be rewritten as “He found diastolic blood pressure and odds of hypertension was significantly raised in the NWO individuals compared with normal weight lean. In addition, adults with NWO had elevated blood glucose and increased odds of high blood sugar levels, highlighting the association between NWO and cardiometabolic derangements.”

Answer: We have made changes in lines 84-88 (current lines of 78-83) of the introduction.

2.Lines 88-90 should be revised to “"People with NWO are likely to develop dyslipidemia [5], insulin resistance [16, 17], changes in blood pressure (BP) [16, 18], and a pro-oxidative status [19]."

Answer: We have made changes in lines 88-90 (current lines of 83-85) of the introduction.

3.In line 95, remove the word ‘in addition’ and start the paragraph with ‘there were few epidemiological studies…’

Answer: We have removed the word ‘in addition’ in line 95 (current line of 91) of the introduction.

4.Lines 494-499 should be rewritten to “Park et al. [41] conducted a genome-wide association study involving 49,915 subjects to identify the NWO genetic indices. He found GCKR, ABCB11, CDKAL1, CDKN2B, NT5C2, and APOC1 gene were associated with metabolically unhealthy phenotypes in individuals with normal weight but not in those with obesity, showing that certain genetic polymorphisms may also have an impact on the metabolic health in NWO populations.”

Answer: We have made changes in lines 494-499 (current lines of 472-474) of the discussion.

5.Lines 518-524 can be rephrased to “Males tend to accrue more visceral fat, leading to the classic android body shape, which has been highly correlated with increased cardiovascular risk. In contrast, females accrue more fat in the subcutaneous depot prior to menopause, a feature that affords protection from the negative consequences associated with obesity and metabolic syndrome [45]. Visceral fat is a source of proinflammatory cytokines that contribute to insulin resistance [46], while the accumulation of fat in the subcutaneous depot is an independent predictor of lower cardiovascular and diabetes-related mortality [47].”

Answer: We have made changes in lines 518-524 (current lines of 491-500) of the discussion.

6.Lines 527-530 could be revised to “Interestingly, FPG, FBG, and HbA1c were not associated with BF%. After adjusting for concomitant variables, we found that NWO was not significantly associated with diabetes in either gender or a specific gender, which was inconsistent with previous findings in China [20, 36]”.

Answer: We have made changes in lines 527-530 (current lines of 501-504) of the discussion.

While revising your submission, please upload your figure files to the Preflight Analysis and Conversion Engine (PACE) digital diagnostic tool, <https://pacev2.apexcovantage.com/>. PACE helps ensure that figures meet PLOS requirements. To use PACE, you must first register as a user. Registration is free. Then, login and navigate to the UPLOAD tab, where you will find detailed instructions on how to use the tool. If you encounter any issues or have any questions when using PACE, please email PLOS at <a href="mailto:figures@plos.org">figures@plos.org. Please note that Supporting Information files do not need this step.

Answer: We have uploaded our figure files to the Preflight Analysis and Conversion Engine (PACE) digital diagnostic tool, and downloaded PACE generated figure files, to ensure our all figures meet PLOS requirements.
